# Surfing the tidal wave: Use of transiently aquatic habitat by juvenile Pacific salmon and other fishes in estuaries

**DOI:** 10.1002/ecy.70100

**Published:** 2025-05-08

**Authors:** Daniel J. Scurfield, Phoebe L. Gross, Julian C. L. Gan, Jonathan W. Moore

**Affiliations:** ^1^ Salmon Watersheds Lab, Earth to Ocean Research Group, Department of Biological Sciences Simon Fraser University Burnaby British Columbia Canada

**Keywords:** climate change, estuary, habitat connectivity, nursery habitat, *Oncorchynchus*, resource waves, restoration

There is an accumulating set of natural history observations of diverse consumers “surfing” resource waves to extend quality foraging opportunities (Armstrong et al., 2016). Resource waves are where natural gradients (e.g., elevation) create pulses of resources that propagate across space and time—thereby extending foraging opportunities for mobile organisms (Aikens et al., 2017). Examples include the seasonal “green wave” for herbivores (Sawyer & Kauffman, 2011), or predators such as grizzly bears (*Ursus arctos*) pursuing sockeye salmon (*Oncorhynchus nerka*) as they migrate to their spawning grounds (Schindler et al., 2013).

While seasonal resource tracking has dominated the literature, tides provide similar opportunities for mobile consumers to exploit ephemeral resources; the framework for the resource wave phenomena (Armstrong et al., 2016). Estuaries are large intertidal landscapes with strong spatiotemporal patterns that typically ebb and flood twice daily across a mosaic of habitats (Figure 1A). Theses habitats range from riparian, transitional marsh, emergent marsh, delta mudflat to eelgrass (*Zostera marina*) providing various forage and shelter opportunities (Woo et al., 2019). While there is general appreciation that estuaries are nursery grounds for juvenile fishes (Sharpe et al., 2019) (Figure 1B), the extent to which these mobile organisms are navigating transiently aquatic habitats remains relatively unknown. Here we ask whether estuarine fishes are accessing new habitats by exploiting tides as a resource wave? Previous studies of juvenile salmon fry have found that they feed heavily on energy‐rich terrestrial insects among estuary marshes potentially mobilized and accessed via tidal inundation (Gray et al., 2002; Woo et al., 2019), while larger predacious fish, such as Dolly Varden (*Salvelinus malma*), occupy deep and large habitats of the outer estuary (Seitz et al., 2020). It is assumed that estuarine fishes will actively seek out newly available forage and cover opportunities while evading the increasing risk of predation as lower estuary habitats gain depth and become increasingly exposed. Therefore, we tested the hypothesis that the abundance of estuarine fish species tracks tidal inundation, moving into transiently aquatic habitat as they become accessible with the use of a series of underwater cameras (Figure 1C).

**FIGURE 1:**
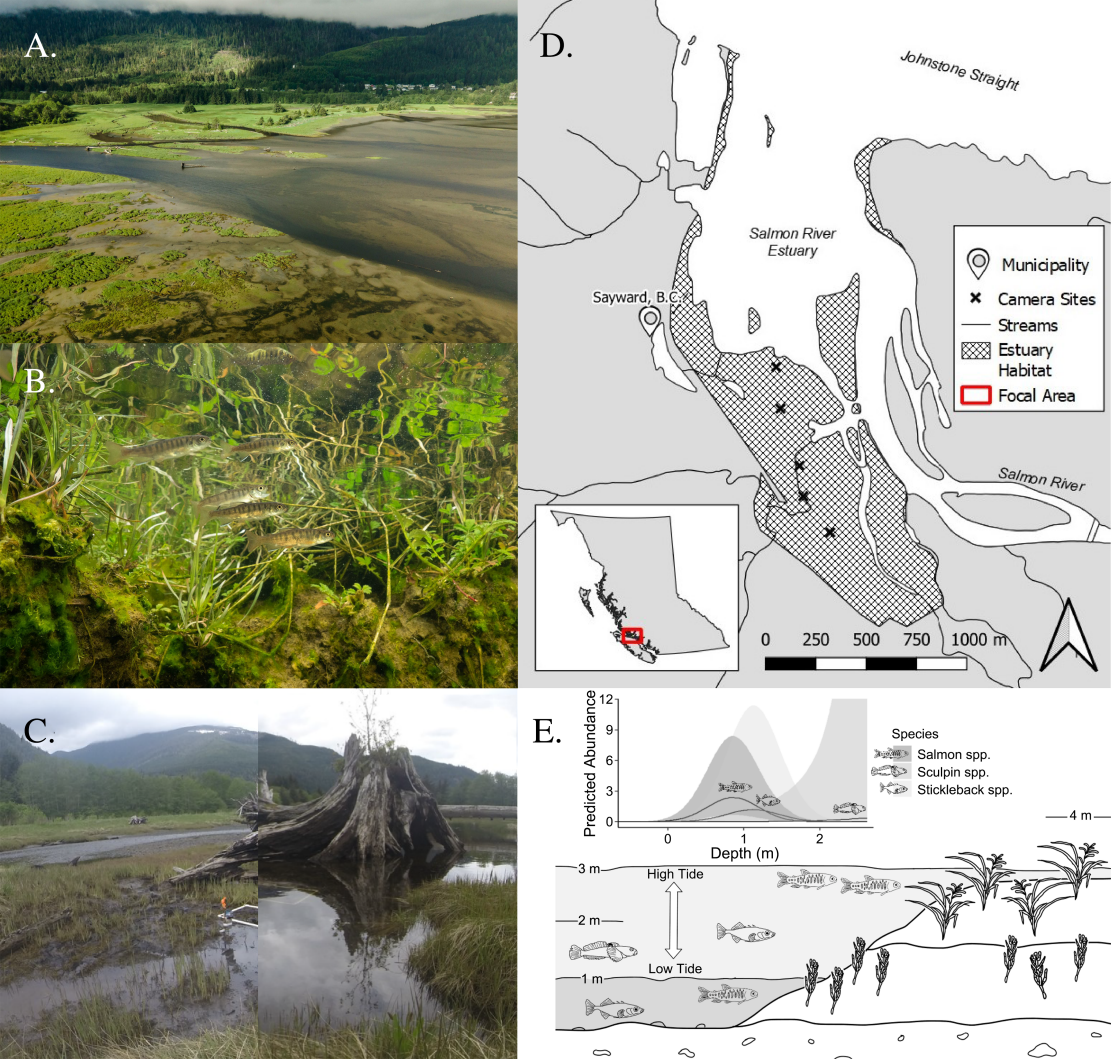
(A) The Salmon (Xwésam) River Estuary transition zone between mudflat and emergent marsh, displaying the diverse ecotones and complex habitat. (B) Coho Salmon (*Oncorhynchus kisutch*) fry utilizing the Salmon River Estuary as stopover habitat during their outmigration to sea. (C) A split image of a camera site located in a channel mid‐estuary at low (left) and high tide (right). (D) Map of the study area. (E) A visualization of salmon, three‐spined stickleback, and sculpin movement across estuary habitat in relation to the tidal stage; inset with a visualization of the model output indicating species mean predicted abundance with 95% CIs. Photographs: Fernando Lessa (A, B), Dan Scurfield (C). Illustrations: Dan Scurfield (E).

This study was conducted in the Salmon (Xwésam) River estuary on the traditional territory of the K'ómoks First Nation, Vancouver Island, British Columbia (Figure 1D). The Salmon River Estuary is a delta estuary approximately 3.5 km^2^ in area, supporting all five species of Pacific salmon and various resident fish species. This work is part of a large‐scale collaborative initiative—Estuary Resilience project—led by The Nature Trust of British Columbia (NTBC), in collaboration with Coastal First Nations and other organizations.

A transect of cameras was established across an elevational gradient in ephemeral tidal channels to capture fish movement (Figure 1D). Each of the five camera sites selected was confirmed to drain completely, providing no fish habitat at low tide. Our sampling window was during peak juvenile salmon abundance in the spring and early summer of 2022, where juvenile Pacific salmon may spend days to months in estuaries during their outmigration (Chalifour et al., 2019; Moore et al., 2016). Video was used to quantify the abundance and composition of fish in the submerged channel habitats, based on passive sampling methods from Ferriss et al. (2021) (Appendix S1: Methods S1). Video was recorded on both flood and ebb tides throughout several tidal cycles during daylight hours during spring and summer 2022. Depth at each site was calibrated from known camera elevation from tide height (Canadian Hydrographic Service, 2022). Timestamped videos were reviewed, identifying fish species to the lowest feasible taxonomic category; salmonids were identified to the genus level, given the variability in phenotypic plasticity of salmon fry.

A generalized mixed‐effects model (GLMM) applying water depth as a predictor for fish abundance and random effect for site, fitted to a zero‐inflated Poisson distribution (marginal Akaike information criterion [mAIC] = 940.7, Appendix S2: Table S1) was selected from the nearest competing model by best fit (ΔmAIC = 13.1, Appendix S2: Table S1) using the mAIC (Greven & Kneib, 2010). Competing models included fixed effect for water depth and habitat, and a random effect for site. Water depth at each site was used as a proxy for tide height to model species abundance across the estuary, standardizing the depth at which habitat becomes accessible. Depth was assigned a quadratic term given the parabolic shape of the abundance data, and fish abundance is constrained to the maximum tide height observed (5.26 m) (Canadian Hydrographic Service, 2022). To evaluate model goodness‐of‐fit, residuals were tested for adequate dispersion (Appendix S3: Figure S1). All analyses were done in the R programming environment, using R version 4.3.1 (R Core Team, 2023), along with packages glmmTMB (Brooks et al., 2017) and DHARMA (Hartig, 2022).

A total of 1408 fish were identified from approximately eight hours of combined underwater footage for a combined total of six tidal cycles. Overall, 557 juvenile Pacific salmon (*Oncorhynchus* spp., fry life‐stage)—114 were confidently identified as coho (*Oncorhynchus kisutch*), 809 three‐spine stickleback (*Gasterosteus aculeatus*), and 39 sculpins (*Cottus* spp.) were identified. Other species including flatfish (*Platichthys stellatus*, *Hippoglossus stenolepis*, and *Parophrys vetulus*), arrow goby (*Clevelandia ios*), and various larval fishes were observed, but in too few numbers to be considered in the model (Scurfield, 2024).

Sequential waves of different fishes used transiently aquatic habitats as tides changed. The first wave was juvenile salmon (*Oncorhynchus* spp.), which entered habitats while it was shallow (~0.31 m) and was predicted to have peak abundance at 0.87 m (Figure 1E) (mean 0.85, SD 0.62). The second wave was three‐spined stickleback (*G. aculeatus*), predicted to have peak abundance at 1.12 m (Figure 1E) (mean 1.08, SD 0.38). Sculpins (*Cottus* spp.) entered near peak tide, with the greatest predicted abundance at a depth of 2.62 m (Figure 1E) (mean 1.32, SD 0.79). The quadratic relationship between depth and abundance was significant for juvenile Pacific salmon (*p* < 0.001) and three‐spined stickleback (*p* < 0.001), implying a strong use of transiently aquatic habitat in intertidal channels when it is temporarily available at an intermediate stage (Figure 1E; Appendix S4: Table S1). Only the linear relationship between depth and abundance was significant for sculpins (*Cottus* spp.) (*p* = 0.046), and the quadratic relationship was insignificant (*p* = 0.375) demonstrating a strong preference for increasingly deep habitats (Figure 1E; Appendix S4: Table S1).

We discovered that estuarine fishes are surfing the tidal wave in estuaries, accessing new habitats using the cyclical momentum of the tides to migrate. In flood tides, otherwise terrestrial habitats periodically transform into aquatic habitats where sequential waves of small fishes enter densely vegetated emergent marshes then return to sparsely vegetated mudflat habitats at low tide. The differential movement between species may be attributed to major differences in body form, swimming ability, forage and cover requirements, or salinity tolerance. However, several videos captured footage of juvenile salmon drift feeding in the emergent marsh, whereas other expected behaviors such as seeking refuge were not observed due to limitations of the study design. Although fish may be using these habitats for a variety of reasons, it is generally understood that habitat selection by juvenile salmon involves species‐specific trade‐offs between optimal foraging opportunities and predation risks (Abrahams & Healey, 1993). Regardless of the ultimate mechanism driving this behavior, this natural history observation illustrates the existence of resource wave surfing operating at the diurnal frequency of the tides, a frequency greater than previous observations of resource surfing (Armstrong et al., 2016).

This study highlights that estuary fishes, including salmon fry, utilize habitats that may be terrestrial (non‐aquatic) for much of the tidal cycle, supporting the assertation that “riparian areas are fish habitat” (Naiman & Latterell, 2005). Similar studies have found that salmon parr‐smolt move tidally in persistently wetted channels in the direction of tidal currents (Hering et al., 2010), yet this study demonstrates successional waves of fish species moving into tidally aquatic habitat. We hypothesize this trend is driven by improved foraging opportunities across species of varying swimming capabilities, though it may also be attributed to a number of environmental and physiological conditions including food web dynamics. Further investigation into forage abundance, fish diet, physiological markers across species, and environmental conditions within estuaries would improve our understanding. In the meantime, aquatic connectivity provides foraging opportunities for resident fish species across various habitats, naturally buffering against climate change impacts such as increased water temperature and “coastal squeeze” due to sea level rise (Borchert et al., 2018; Fulford et al., 2014). Estuarine connectivity continues to enable mobile consumers to access diverse resources and adapt to varying environmental conditions.

## CONFLICT OF INTEREST STATEMENT

The authors declare no conflicts of interest.

## Supporting information


Appendix S1:

Appendix S2:

Appendix S3:

Appendix S4:

## Data Availability

Data (Scurfield, 2024) are available in Zenodo at https://doi.org/10.5281/zenodo.14026634.
