# Supplementary material for: Surfing the tidal wave: Use of transiently aquatic habitat by juvenile Pacific salmon and other fishes in estuaries
Source: Ecology. 2025 May 8;106(5):e70100. doi: 10.1002/ecy.70100 (PMC12060844; doi:10.1002/ecy.70100)
Supplement: Supplementary file 1 — Appendix S1: [file ECY-106-e70100-s003.pdf]

Ecology. Daniel J. Scurfield, Phoebe L. Gross, Julian C.L. Gan, and Jonathan W. Moore. Surfing the tidal wave: Use of transiently-aquatic habitat by juvenile Pacific salmon and other fishes in estuaries.

Appendix S1: Methods S1

Underwater camera specifications

We used underwater video (GoPro Hero 3+ and Hero 4 mounted with a CamDo Blink timelaspse controller) to quantify abundance and composition of fish in the submerged intertidal channel habitats, based on methods from Ferriss et al., 2021 and Phillips et al., 2022. Cameras housed in a waterproof case were mounted to ½” painted rebar and installed at each site 15 cm above the substrate at 15˚ downward angle. A PVC quadrat (50 cm^2) was secured to the substrate 30 cm in front of the camera, to provide a standardized field of view across sites. Video was recorded for 1 min on 10 min intervals over a period of 4 h. This was performed for several tidal cycles during daylight hours throughout late spring and early summer 2022 (May 1, 15, and 17, June 7 and 8, and July 2, 2023), the period when juvenile salmon are most abundant in estuaries of Vancouver Islands coastal streams (Holtby and Scrivener, 1989). With cameras facing downstream, video was collected on both flood and ebb tides. Site depth was determined by subtracting the tide height (Canadian Hydrographic Service, 2022) from the known camera elevation at each site.

These sites range from low to high channel elevation of 1.90 m to 3.45 m from chart datum (i.e., sea level or 0 m elevation). Each camera site is approximately 100-150 m apart, and placed within proximity (<2 m) of available cover structures (i.e., large woody debris (LWD) and undercut banks), known to provide cover for juvenile salmon (Hafs et al., 2014). The lowermost site was characterized by sparsely vegetated (e.g., *Fucus spp*., and *Zostera spp*.) wide (>20 m) and shallow (<1 m) mudflat channel and transitioned to a narrow (<1m), shallow (<1m), and densely vegetated (e.g. *Salix spp.*, *Fritillaria affinis*, *Potentilla anserina, etc.*) channel of the uppermost site. This method provides an alternative to quantify fish movement and behavior in complex or inaccessible habitats at life-stages too small for feasible telemetry studies, while avoiding potential sampling bias of other methods (i.e., seining, snorkel surveys).

Literature Cited

The Canadian Hydrographic Service. (n.d.). *Kelsey Bay (08215) tide predictions*. Retrieved August 1, 2022, from https://www.tides.gc.ca/en/stations/8215

Ferriss, B., Veggerby, K., Bogeberg, M., Conway-Cranos, L., Hoberecht, L., Kiffney, P., Litle, K., Toft, J., & Sanderson, B. (2021). Characterizing the habitat function of bivalve aquaculture using underwater video. *Aquaculture Environment Interactions*, *13*, 439–454. https://doi.org/10.3354/aei00418

Hafs, A. W., Harrison, L. R., Utz, R. M., and Dunne, T. (2014). Quantifying the role of woody debris in providing bioenergetically favorable habitat for juvenile salmon. *Ecological Modelling, 285,* 30–38.

Phillips, G., Redman, D., Schultz, K., Mercaldo-Allen, R., & Rose, J. M. (2022). Using underwater video to observe aquaculture gear in Long Island Sound—A citizen science guide. NOAA Fisheries, Northeast Fisheries Science Center, Milford Laboratory.
