# Supplementary material for: Surfing the tidal wave: Use of transiently aquatic habitat by juvenile Pacific salmon and other fishes in estuaries
Source: Ecology. 2025 May 8;106(5):e70100. doi: 10.1002/ecy.70100 (PMC12060844; doi:10.1002/ecy.70100)
Supplement: Supplementary file 2 — Appendix S2: [file ECY-106-e70100-s004.pdf]

Ecology. Daniel J. Scurfield, Phoebe L. Gross, Julian C.L. Gan, and Jonathan W. Moore. Surfing the tidal wave: Use of transiently-aquatic habitat by juvenile Pacific salmon and other fishes in estuaries.

Appendix S2: Table S1

Table S1. Summary of General Linear Mixed Effects model selection. Random effect (RE) noted.

**Model Covariates**	**mAIC**	**Δ mAIC**	**BIC**	**Deviance**	**DF**
Depth + Site (RE)	940.7	0	967.9	934.7	5
Depth + Habitat + Site (RE)	953.8	13.1	994.2	947.8	7
Depth	1073.8	133.1	1094.3	1067.8	4
Depth + Habitat	1156.8	216.1	1190.6	1150.8	5
