# Supplementary material for: Surfing the tidal wave: Use of transiently aquatic habitat by juvenile Pacific salmon and other fishes in estuaries
Source: Ecology. 2025 May 8;106(5):e70100. doi: 10.1002/ecy.70100 (PMC12060844; doi:10.1002/ecy.70100)
Supplement: Supplementary file 3 — Appendix S3: [file ECY-106-e70100-s002.pdf]

Ecology. Daniel J. Scurfield, Phoebe L. Gross, Julian C.L. Gan, and Jonathan W. Moore. Surfing the tidal wave: Use of transiently-aquatic habitat by juvenile Pacific salmon and other fishes in estuaries.

Appendix S3: Figure. S1

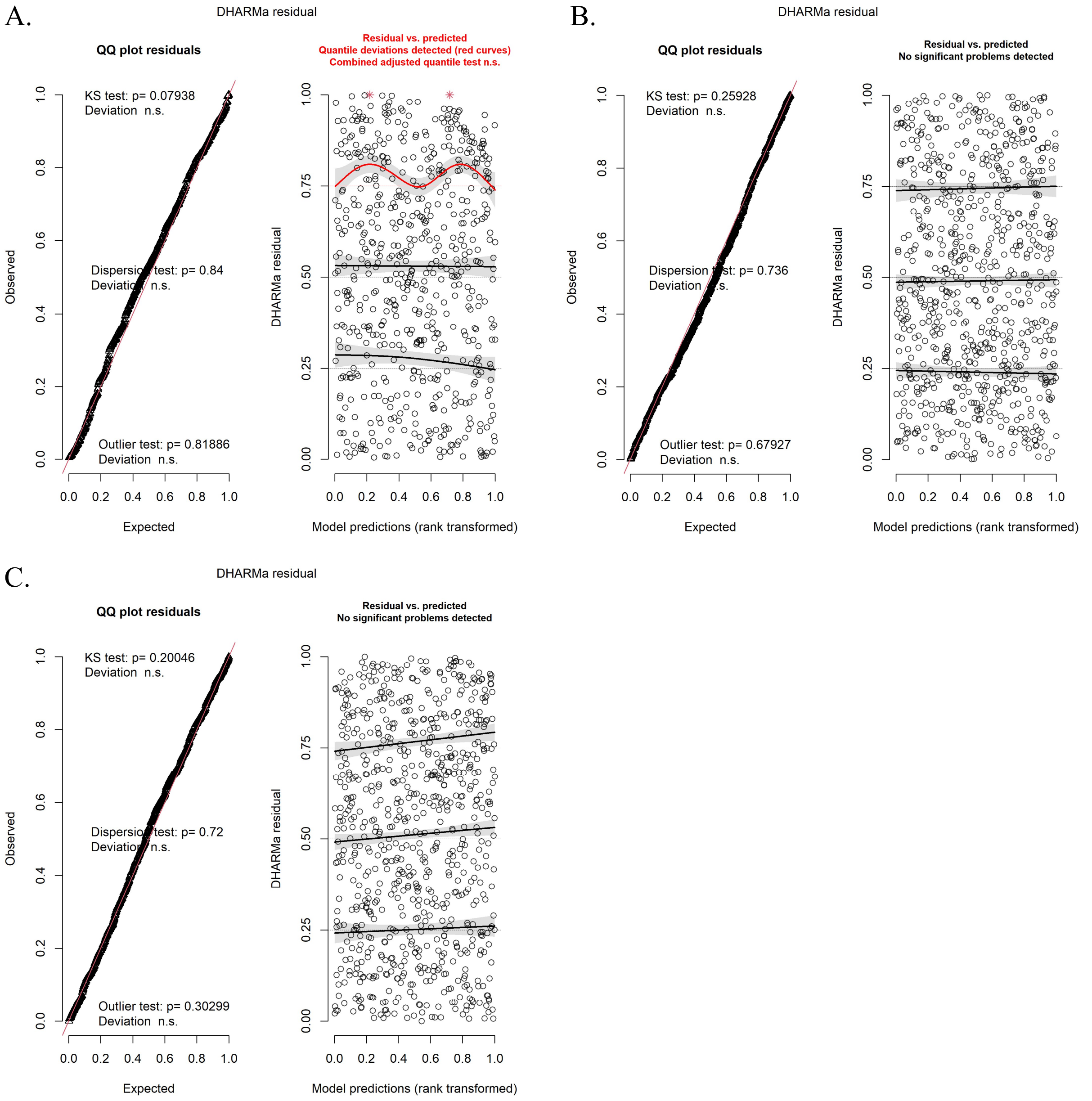

Figure S1. (A) Residual diagnostics for GLMM applied to Pacific Salmon abundance. (B) Residual diagnostics for GLMM applied to three-spined stickleback abundance. (C) Residual diagnostics for GLMM applied to sculpin abundance.
