# Supplementary material for: Surfing the tidal wave: Use of transiently aquatic habitat by juvenile Pacific salmon and other fishes in estuaries
Source: Ecology. 2025 May 8;106(5):e70100. doi: 10.1002/ecy.70100 (PMC12060844; doi:10.1002/ecy.70100)
Supplement: Supplementary file 4 — Appendix S4: [file ECY-106-e70100-s001.pdf]

**Ecology**. Daniel J. Scurfield, Phoebe L. Gross, Julian C.L. Gan, and Jonathan W. Moore. Surfing the tidal wave: Use of transiently-aquatic habitat by juvenile Pacific salmon and other fishes in estuaries.

**Appendix S4: Table S1**

Table S1. General Linear Mixed Effects model summary for study species. The explanatory variable Depth is the linear relationship and $Depth^2$ is the parabolic relationship with species abundance.

| | Pacific salmon (juvenile) *Onchorynchus spp.* | | | Three-spined stickleback *Gasterosteus aculeatus* | | | Sculpin species *Cottus spp.* | | |
|---|---|---|---|---|---|---|---|---|---|
| **Explanation** | **Estimate** | **SE** | ***p*** | **Estimate** | **SE** | ***p*** | **Estimate** | **SE** | ***p*** |
| **Fixed Effects** | | | | | | | | | |
| (Intercept) | -8.40 | 1.53 | **<0.001** | -7.91 | 1.43 | **<0.001** | -6.42 | 1.87 | **<0.001** |
| Depth | 270.45 | 44.66 | **<0.001** | 247.64 | 36.96 | **<0.001** | 92.46 | 46.42 | **0.046** |
| $Depth^2$ | -159.54 | 29.10 | **<0.001** | -125.39 | 20.16 | **<0.001** | -19.14 | 21.59 | 0.375 |
